# Curricular priorities for business ethics in medical practice and research: recommendations from Delphi consensus panels

**DOI:** 10.1186/1472-6920-14-235

**Published:** 2014-11-15

**Authors:** James M DuBois, Elena M Kraus, Kamal Gursahani, Anthony Mikulec, Erin Bakanas

**Affiliations:** Center for Clinical Research Ethics, Division of General Medical Sciences, Washington University School of Medicine, 660 S. Euclid Ave, Saint Louis, MO 63110 USA; Bander Center for Medical Business Ethics, Saint Louis University, Salus Center, 3545 Lafayette Ave, Saint Louis, MO 63104-1314 USA; Department of Surgery, Division of Emergency Medicine, Saint Louis University School of Medicine, 3635 Vista Avenue at Grand Blvd, Saint Louis, MO 63110 USA; Department of Otolaryngology-Head and Neck Surgery, Saint Louis University School of Medicine, 3635 Vista Avenue at Grand Blvd, Saint Louis, MO 63110 USA; Department of Internal Medicine, Division of General Internal Medicine, 12 South FDT, Saint Louis University School of Medicine, 1402 S. Grand Blvd, Saint Louis, MO 63104 USA

**Keywords:** Medical business ethics, Professional ethics, Clinical ethics, Medical ethics, Medical education, Graduate medical education, Business in medicine, Healthcare industry, Delphi consensus

## Abstract

**Background:**

No published curricula in the area of medical business ethics exist. This is surprising given that physicians wrestle daily with business decisions and that professional associations, the Institute of Medicine, Health and Human Services, Congress, and industry have issued related guidelines over the past 5 years. To fill this gap, the authors aimed (1) to identify the full range of medical business ethics topics that experts consider important to teach, and (2) to establish curricular priorities through expert consensus.

**Methods:**

In spring 2012, the authors conducted an online Delphi survey with two heterogeneous panels of experts recruited in the United States. One panel focused on business ethics in medical practice (n = 14), and 1 focused on business ethics in medical research (n = 12).

**Results:**

Panel 1 generated an initial list of 14 major topics related to business ethics in medical practice, and subsequently rated 6 topics as very important or essential to teach. Panel 2 generated an initial list of 10 major topics related to business ethics in medical research, and subsequently rated 5 as very important or essential. In both domains, the panel strongly recommended addressing problems that conflicts of interest can cause, legal guidelines, and the goals or ideals of the profession.

**Conclusions:**

The Bander Center for Medical Business Ethics at Saint Louis University will use the results of the Delphi panel to develop online curricular resources for each of the highest rated topics.

**Electronic supplementary material:**

The online version of this article (doi:10.1186/1472-6920-14-235) contains supplementary material, which is available to authorized users.

## Background

A recent Institute of Medicine report estimates that annually up to 30% ($765 billion) of health care costs are potentially avoidable: nearly $210 billion may be attributable to unnecessary services provided by physicians and $55 billion attributable to missed prevention opportunities [1]. Against this background, the Alliance for Academic Internal Medicine and the American College of Physicians collaborated in producing a curriculum for residents focused on incorporating high-value, cost-conscious care principles into their clinical practice [2]. The development of this curriculum can be seen as part of a larger movement within academic medical centers to educate physicians about the business dimension of medicine. Several model curricula have been published over the past 5 years addressing a wide variety of topics [2–6]. Nevertheless, preliminary research reveals that, while conflicts of interest are sometimes addressed, there are no proposed curricula in the larger topic of medical business ethics, which we define as the ethical engagement of the financial dimension of medical practice and research. Many important topics such as Medicare fraud and abuse are not uniformly presented to training physicians in the United States [6].

This is surprising for several reasons. First, financially motivated violations of professional ethics in medical practice and research have received growing attention in the media, including the performance of unnecessary surgeries with the aim of increasing income, upcoding, and conducting research without adequately disclosing industry sponsorship [7–10]. Preserving trust in the institution of medicine will require interventions to protect medical professionalism in an increasingly complex business environment. Second, over the past 5 years, professional associations [11, 12], the Institute of Medicine [13], the Department of Health and Human Services [14], Congress [15], and the pharmaceutical industry [16], have issued new guidelines and requirements addressing financial conflicts of interest in medical practice and research. It will be important to update the curricula of medical ethics and professionalism training programs to incorporate information about these evolving standards and requirements. Third, the Accreditation Council for Graduate Medical Education (ACGME) lists both professionalism (which includes “an adherence to ethical principles”) and systems-based practice (which includes “an awareness of the larger context and system of health care”) as two of their 6 core competencies of patient care [17]. Moreover, medical students and residents support initiatives in ethics and professionalism educational initiatives [18]. Nevertheless, while courses in medical ethics have been an established part of the medical curriculum for more than a decade [19, 20], these courses have traditionally focused on matters of clinical ethics with limited regard for the impact financial factors can have on clinical care. Finally, physicians struggle with questions of business ethics on a day-to-day basis as they decide whether to use newer more expensive treatments, respond to demands from hospital administrators and practice managers, collaborate with device representatives, or decide whether to enroll a patient in a clinical trial [21, 22].

The Bander Center for Medical Business Ethics was established at Saint Louis University with an endowment gift from the BF Charitable Foundation in 2007 with the mission of promoting ethical business practices in medical care and research through the development of training and investigation opportunities for medical students, residents and physicians in practice. Consistent with this mission, Bander Center-affiliated faculty and staff collaborated in conducting a Delphi consensus panel project to establish priorities for curricula in business ethics in medical practice and research. Delphi panels are used with the purpose of establishing a consensus on topics such as health care quality indicators [23], policy priorities [24], or educational curricula [25]. Often the purpose is to establish priorities in the face of an overwhelming number of options (such as quality indicators or curricular topics). However, given the relative lack of any guidance on developing curricula in medical business ethics, our Delphi project had two aims: (a) to identify the full range of medical business ethics topics that experts generally consider important to teach (that is, to sketch the landscape of medical business ethics); and (b) to establish curricular priorities through expert consensus.

## Methods

### Project design and panelists

Two national Delphi consensus panels were conducted by the Bander Center for Medical Business Ethics at Saint Louis University using online surveys hosted in Qualtrics, which is an online survey software system that is HIPAA compliant. (The software system is available from Qualtrics LLC, Provo, UT through http://www.qualtrics.com, site accessed August 13, 2014.) Participants accessed the survey through unique links, and survey administrators accessed data via a password protected website. Participation was identifiable only to the survey administrators (the first two authors), which enabled us to follow up with targeted reminders across both rounds of the survey.

Delphi panels are typically conducted using surveys to enable anonymous, individual responses, which prevents groupthink [26]. Delphi panels begin by posing an open-ended question (round 1); the project team then collates and analyzes round 1 responses to produce clear lists of items. In round 2, the project team presents panelists with refined responses from round 1, asking them to rate items using a Likert-type scale. “Consensus” may be defined in a variety of ways, either by requiring a majority or super-majority of respondents to score an item as high-priority or by using a mean score as a cut off point. A third round may be used to refine answers when new information is required [27].

Our purpose in using a Delphi survey was not to produce generalizable knowledge, but rather to identify consensus among experts. Accordingly, panelists were selected using purposive, non-probability sampling. Delphi panels typically include experts in a field [26, 27]. We aimed to recruit heterogeneous experts on our panels for two reasons. First, panels that include diverse stakeholders—such as physicians in training, physicians in practice, researchers, administrators, and government oversight personnel—enjoy increased credibility and acceptance [23]. Second, because business ethics in medical research and practice is such a young area of investigation, few well-rounded experts exist. It is therefore necessary to recruit people with diverse areas of specialization in order to represent the full range of issues in medical business ethics.

Panelists were identified through searches of PubMed and Google using relevant keywords such as “medical business ethics, “conflicts of interest,” “ethics and fraud,” as well as personal contacts of team members, who have actively studied medical business ethics as fellows, staff, or faculty within the Bander Center for Medical Business Ethics at Saint Louis University. Panelists received an email invitation that provided details on the topic, process, and time commitment. Additionally, one member of the authorship team participated on each panel (DuBois on the research panel, and Bakanas on the practice panel). This enabled each of the two authors to contribute topics to the first round based on their literature reviews and general expertise; however, the potential to bias outcomes was limited by three facts: On each panel 92% or more of the panelists were not authors, each panelist provided responses independently, and panelists were blind to identity of other panelists.

Panel 1 addressed curricular priorities for education on business ethics in medical *practice*. Panelists are listed in (Additional file 1), and included individuals with expertise in medical practice, medical education, medical ethics, medical sociology, health care administration, health economics, health law, outcomes research, and government oversight. The panel had 14 experts; 12 participated in both rounds.

Panel 2 addressed business ethics in medical *research*. Panelists are listed in (Additional file 2) and included individuals with expertise in medical research, research training, research ethics, social science, research administration, health economics, research regulations, and government oversight. Panel 2 had 12 experts; 10 participated in both rounds. Both participation rates across the two rounds are excellent [26]. Round 1 began in spring 2012; round 2 began in summer 2012 and closed in August.

A description of the consensus-building project was provided to the Institutional Review Board (IRB) at Saint Louis University. The corresponding author received a determination letter from the IRB stating that the project did not constitute research because its purpose was to generate a consensus rather than generalizable knowledge. However, all participants freely consented to participate in the panel and provided permission to publish their names and biographical details, which they reviewed and approved.

### Survey prompts and response analysis

In Round 1 participants responded to the following prompt:

Please list up to 10 topics that you consider most important to address within educational programs for physicians-in-training in the domain of business ethics in medical practice [panel 1]/research [panel 2].

[For panel 1] Your topics may pertain to matters of law, reimbursement, ethics, professionalism, and any other issues you find most relevant.

[For panel 2] Your topics may pertain to matters of law, research funding, ethics, professionalism, and other matters you find most relevant.

To prepare items for Round 2, the project team: (1) collated all Round 1 responses; (2) eliminated responses that were unclear (such as Program Integrity), unrelated to business ethics (such as Euthanasia in Europe or Genetic Testing), or redundant; and (3) identified major subject headings and subsumed specific topics under them.

In Round 2 the project team presented panelists with each distinct major topic with 2 – 3 examples of subtopics that might be addressed within educational sessions. Panelists were asked to rate separately how important they think it is to teach each of the topics to medical students and to residents: (1) not at all important; (2) somewhat important; (3) important; (4) very important; or (5) essential.

We defined a consensus on the importance of teaching a topic as receiving a score of 4 (very important) or 5 (essential) from a simple majority of panelists. Sometimes a higher threshold is used to define a consensus (such as a super majority of 70% or higher); but the research team deemed this inappropriate as most experts on our panel had expertise on only some topics relevant to medical business ethics.

## Results

### Results from Panel 1: business ethics in medical practice

In response to our initial open-ended prompt, panel 1 produced 103 total responses. The project team reduced these to 14 distinct major topics, each with 2 – 3 subtopics. Table 1 presents all 14 topics from round 1.

**Table 1:** **Curricular priorities for business ethics in medical practice**

Topic	Consensus for MS Curriculum	Mean for MS Curriculum	Consensus for PG Curriculum	Mean for PG Curriculum
Problems that can arise from conflicts of interest	**YES (10/12)**	**4.4**	**YES (9/12)**	4.6
- Biased prescribing, advising on formularies, or selection of devices				
- Harm to patient trust				
- Bias operates unconsciously and unintentionally, making it difficult to manage				
General healthcare organization and systems	**YES (8/12)**	**4.1**	**YES (11/12)**	4.4
- Medicaid/Medicare and private insurance industry				
- Drivers of cost increases, gaps in system, and other challenges				
- Alternative models, including international models				
Fostering patient care quality and safety	**YES (9/12)**	**4.1**	**YES (10/12)**	4.3
The cost of medical errors				
- Strategies for improving patient care quality				
- Strategies for addressing medical errors				
Medical professionalism, the goals of medicine, and their relationship to medical ethics	**YES (7/12)**	**3.9**	**YES (8/12)**	4.0
- Primacy of patient well-being in physician-patient relationship; fiduciary obligations				
- Balancing secondary gains (to finances, career, or life-work balance) with obligations to patients				
- Physicians as advocates for system change				
The structure and ethical issues surrounding reimbursement systems for physicians	**YES (7/12)**	**3.8**	**YES (10/12)**	4.5
- Pay for performance				
- Fee for service				
- Managed care and capitated payment systems				
Conflicts of interest arising from physician relationships with pharmaceutical and device industries	NO (6/12)	3.8	**YES (8/12)**	4.2
- Free samples as marketing				
- Consulting relationships and speakers bureaus				
- Sponsoring CME				
The legal framework for the business of medicine	YES (7/12)	3.7	**YES (9/12)**	4.2
- False claims act, anti-kick back statute, Stark self-referral law				
- Possible penalties and sanctions				
- How law influences behavior				
Good stewardship in resource utilization	NO (6/12)	3.6	**YES (9/12)**	4.2
- Exploring costs of competing treatment options				
- Rationing strategies				
- Value of stewardship when resources are limited				
Oversight of the practice of medicine	NO (5/12)	3.1	NO (6/12)	3.5
- Self-regulation vs. government regulation				
- Whistleblowing—mechanics, risks, protections, responsibilities				
The business relationships of academic medical centers - Influence of educational and research mission on patient care	NO (3/12)	2.9	NO (5/12)	3.5
- Financial pressures on academic medical centers and influence on medical practice				
- Advantages and disadvantages of specific business relationships of academic medical centers				
Disclosure rules and strategies for managing conflicts of interest	NO (2/12)	2.8	NO (5/12)	3.6
- Disclosure rules such as the Physician Payments Sunshine Act and institutional policies				
- Strategies such as divestment or increased oversight				
Resources physicians can consult on matters of business ethics and compliance	NO (2/12)	2.7	NO (5/12)	3.4
- Institutional resources, including compliance officers				
- External resources, including Bar Association, health lawyers, CMS medical directors				
- Educational materials, including Officer of Inspector General and Institute of Medicine publications				
Mechanics of documentation, coding, billing and audits	NO (2/12)	2.6	NO (5/12)	3.6
- Accurate billing vs. upcoding				
- Documentation—the need and best practices				
Physician ownership of practices and facilities	NO (2/12)	2.6	NO (5/12)	3.7
- Advantages and disadvantages of ownership				
- Avoiding violations of self-referral and kick back laws				
Conflicting interests arising from ownership vs from working as employee or consultant				

Legend:

- MS = Medical Student.

- PG = Post Graduate or Resident.

Notes:

- Consensus defined as >50% of panelists (n = 12) rated item as “very important” or “essential” (the top 2 of 5 ratings) in round 2. Results reflecting consensus appear in boldface.

- Topics listed in rank order using the MS curriculum mean scores.

- Mean scores are based upon a 5-point Likert-type scale.

- Beneath each major topic heading above, we list the bulleted subtopics that were presented along with the overarching topics that were rated. Many subtopics were based on topics presented in round 1; some were added by the project team prior to round 2

- Bullets indicate subtopics that were presented along with the overarching topics that were rated. Many subtopics were based on topics presented in round 1; some were added by the project team prior to round 2.

A majority of panelists rated 6 topics as very important or essential to teach to medical students and residents: (1) problems that can arise from conflicts of interest; (2) general healthcare organization and systems; (3) fostering patient care quality and safety; (4) medical professionalism, the goals of medicine, and their relationship to medical ethics; (5) the structure of and ethical issues surrounding reimbursement systems for physicians; and (6) the legal framework for the business of medicine. Table 1 presents these topics rank ordered using the mean medical student (MS) curriculum importance score; it also presents subtopics within each of these categories. Two additional topics received a majority rating of 4 or 5 for residents or post-graduates (PGs): (6) conflicts of interest arising from physician relationships with pharmaceutical and device industries; and (8) good stewardship and resource utilization. The remaining topics received a mean rating of less than 4 for both educational groups and failed to achieve a consensus for either group.

### Results from Panel 2: business ethics in medical research

Panel 2 generated 97 total responses. These were reduced to 10 distinct major topics, each with 2 – 3 subtopics. Table 2 presents the 10 topics from round 1.

**Table 2:** **Curricular priorities for business ethics in medical research**

Topic	Consensus for MS Curriculum	Mean for MS Curriculum	Consensus for PG Curriculum	Mean for PG Curriculum
The ideals of the medical research profession	**YES (8/10)**	**4.3**	**YES (8/10)**	**4.2**
- Pursuing new knowledge				
- Developing new drugs and devices to serve patients				
- Protecting the welfare of human and animal subjects				
Potential problems that conflicts of interest cause	**YES (8/10)**	**4.2**	**YES (10/10)**	**4.5**
- Inappropriate participant recruitment				
- Biased data publication and ghost authorship				
- Shifting research priorities				
Strategies for managing conflicts of interest in research	**YES (8/10)**	**4.2**	**YES (9/10)**	**4.4**
- Disclosure				
- Increased oversight				
- Divestment or recusal from specific roles				
Challenges of playing the roles of both physician and researcher	**YES (7/10)**	**4.0**	**YES (10/10)**	**4.5**
- Possibility of ‘therapeutic misconception’—when patients mistake research participation for individualized therapy				
- Conflicting roles could contribute to tacit pressure on patients to enroll or to biased presentation of consent information				
Legal and policy issues surrounding conflicts of interest in research	**YES (6/10)**	**3.8**	**YES (8/10)**	**4.4**
- NIH conflict of interest policies				
- Institutional policies				
- Bayh-Dole act				
Valid clinical research study design	NO (5/10)	3.7	**YES (6/10)**	**4.0**
- Good research practices				
- Common deviations from good research practices				
Issues in academic medical research centers	NO (2/10)	3.0	NO (4/10)	3.3
- Pressures to obtain grant and contract funding				
- Pressures to publish				
- Balancing roles as investigator and mentor/educator				
Physician as entrepreneur, patent holder, and owner of data and materials	NO (2/10)	2.7	NO (4/10)	3.3
- Federal and institutional rules on patents and data ownership				
- Managing conflicts of interest in ‘start up’ company research				
- Managing institutional conflicts of interest				
Institutional offices that provide information and oversight on fiscal matters in research	NO (3/10)	2.7	NO (5/10)	3.3
- Office of research services (or pre-award program)				
- Office of sponsored programs (or post-award program)				
- Conflict of interest committee				
- Research integrity office				
Research budgeting, costs, and billing	NO (2/10)	2.5	NO (3/10)	3.0
- OMB circular A-21 rules on allowable costs				
- Effort reporting and conflicts of commitment				
- Accurate budget development				

Legend:

- MS = Medical Student.

- PG = Post Graduate or Resident.

Notes:

- Consensus defined as >50% of panelists (n = 10) rated item as “very important” or “essential” (the top 2 of 5 ratings) in round 2. Results reflecting a consensus appear in boldface.

- Topics listed in rank order using the MS curriculum mean scores.

- Mean scores are based upon a 5-point Likert-type scale.

- Beneath each major topic heading above, we list the bulleted subtopics that were presented along with the overarching topics that were rated. Many subtopics were based on topics presented in round 1; some were added by the project team prior to round 2.

A majority of panelists rated 5 topics as very important or essential to teach to medical students and residents: (1) the ideals of the medical research profession; (2) potential problems that conflicts of interest cause; (3) strategies for managing conflicts of interest in research; (4) challenges of playing the roles of both physician and researcher; and (5) legal and policy issues surrounding conflicts of interest in research. Table 2 presents these topics rank ordered using the mean MS curriculum importance score; it also presents subtopics within each of these categories. One additional topic received a majority rating of 4 or 5 for PGs: (6) valid clinical research study design. The remaining topics received a mean rating less of than 4 for both educational groups and failed to achieve a consensus for either group.

## Discussion

The Delphi panels achieved our two aims: (a) to identify the full range of medical business ethics topics that experts generally consider important to teach (that is, to sketch the landscape of medical business ethics); and (b) to establish curricular priorities through expert consensus. On the one hand, Tables 1 and 2 identify a broad range of topics, and no topic in medical research or practice received a mean score of less than 3 (important) for resident education. That is, both lists are comprised of topics that are relevant to medical business ethics and important to address prior to practicing medicine independently. The entirety of both tables may be of value in educating residents; shadings and rankings may help prioritize topics when curricular time is tight. On the other hand, Tables 1 and 2 establish curricular priorities by identifying a top-5 list in each domain.

The project team believes that the two tables list topics that accurately reflect the scope of the field of medical business ethics as it is currently represented in the medical and ethics literature and health law.

We were somewhat surprised that some topics were not identified as curricular priorities; for example, strategies for managing conflicts of interest in medical practice and the identification of institutional offices that provide information and oversight on fiscal matters in research. However, as noted already, no topic received a mean score lower than 3 (important) for medical residents; thus, failure to make our “top 5” list does not indicate a topic is unimportant for practicing physicians.

More importantly, we were pleased that both panels established as a priority exploring the fundamental goals and ideals of the profession. While it may not be immediately apparent that topics such as “the goals of medicine and their relation to medical ethics” or “the ideals of the medical research profession” directly relate to medical business ethics, a strong commitment to the goals of medical practice and research may do more to guide physicians in their business practices than reminding them of specific rules, which are granular and subject to ongoing change. Given that the whole problem of conflicts of interest is that they provide a motive (consciously or unconsciously) to prioritize personal interest above the primary goals of medicine or research, exploring the significance of these goals makes good sense [28]. As we write this, we assume that the primary goal of medicine is patient care focused on prevention, healing, and palliation, and that the medical relationship is a fiduciary relationship in which patient interests must take priority over other interest, such as profit, research, and education, which are legitimate, but not primary within the context of a patient-physician relationship [28].

We also believe it is wholly appropriate that a greater number of topics were identified as important for medical residents than medical students, given that medical students frequently lack the relevant experience to make the topics salient, and they are not yet “stakeholders” in the truest sense given that they do not bill for services and only rarely serve as principal investigators.

In undertaking this project, we have made no assumptions regarding where such material might best be taught. Dealing well with business matters in medical practice is an essential part of medical ethics and medical professionalism; embedding medical business ethics in such courses or into courses on the healthcare delivery system would make good sense. Similarly, business ethics in research may be addressed effectively by expanding the focus of current research ethics courses, many of which already address conflicts of interest. However, concurrent with the Delphi survey reported in this paper, project team members simultaneously conducted a survey of medical students and residents at two Mid-western schools of medicine, which established a need for and interest in receiving training in medical business ethics [29]. In that survey we identified strong interest in “background” issues such as the structure of the healthcare system and reimbursement systems; accordingly, it may be most effective to integrate discussion of ethics cases into units that address these larger business issues. If faculty members are provided with discussion guides, it may be quite feasible to integrate discussion of medical business ethics cases into individual class sessions or “lunch and learn” sessions with residents. In this manner, we believe it is realistic to cover the 5 top-rated topics across the 4 years of medical school or during residency programs.

### Limitations

This project has several limitations. First, our purpose was to identify a consensus among a nonprobability sample of experts. It cannot be assumed that the consensus among our groups of 12 – 14 experts would be identical to the consensus among a different group of experts. Second, given the relative novelty of the area of medical business ethics, few well-rounded experts exist. Our panelists all represented a stakeholder group or expert group of interest; but most have narrow areas of expertise. Third, we focused on identifying curricular topics without conducting additional Delphi rounds to identify the level of mastery needed. We did this because the topics are so diverse that the meaning of “basic” versus “advanced” mastery is not constant. At one extreme, advanced mastery of the healthcare delivery system might require an advanced degree; at the other extreme one could teach the identification of local resources for medical business ethics after memorizing a 1-page handout—an “advanced” level of mastery not much greater than “basic.”

### Next steps

The Bander Center for Medical Business Ethics at Saint Louis University has used both Delphi panels’ list of curricular priorities to develop a casebook in medical business ethics for medical students and residents, consisting of 14 cases followed by fact sheets, a list of relevant ethical principles, and a presentation of relevant U.S. laws. The casebook is indexed to the Delphi topics, and at least one case engages each topic. The casebook and other supporting materials will be available online in September 2014 at http://www.slu.edu/bander-center-home/resources. Given the nascent state of the field of medical business ethics, the team is committed to developing collections of relatively few, high-quality materials rather than developing comprehensive repositories of materials. We believe this will be more useful to instructors or mentors who themselves may lack extensive mastery of the broader subject matter.

## Conclusions

Medical business ethics has attracted increasing attention in recent years and deserves to be addressed explicitly in undergraduate and post-graduate medical education. The number and breadth of relevant topics can seem overwhelming particularly in the face of limited educational time and numerous competing curricular priorities; however, the top 5 topics identified in the domains of medical practice and research lend themselves to exploratory presentations using diverse formats. More importantly, several high-priority topics might be covered in other contexts (such as healthcare organization or patient care quality and safety), reinforcing the point that medical business ethics is simply a dimension of the good practice of medicine in today’s complex healthcare system.

## Electronic supplementary material

Additional file 1: **Business Ethics in Medical Practice Panelists.** (DOC 35 KB)

Additional file 2: **Business Ethics in Medical Research Panelists.** (DOC 36 KB)
