# Supplementary material for: Curricular priorities for business ethics in medical practice and research: recommendations from Delphi consensus panels
Source: BMC Med Educ. 2014 Nov 15;14:235. doi: 10.1186/1472-6920-14-235 (PMC4289297; doi:10.1186/1472-6920-14-235)
Supplement: Supplementary file 1 — Additional file 1: Business Ethics in Medical Practice Panelists. (DOC 35 KB) [file 12909_2013_1100_MOESM1_ESM.doc]

Supplemental Content

**Appendix A: Business Ethics in Medical Practice Panelists**

**Erin Bakanas, MD, MAHCM**

[bakanase@slu.edu](mailto:ebakanas@slu.edu)

Dr. Bakanas received her MD from the University of Connecticut School of Medicine and Dentistry and her MA in Health Care Mission from the Aquinas Institute of Theology in Saint Louis. She is currently an Associate Professor in the Department of Internal Medicine at Saint Louis University and Interim Director of the Bander Center for Medical Business Ethic. She is highly involved with teaching and mentoring activities at the school of medicine, including co-directing an elective entitled “Who’s Buying Lunch?” on physician-pharmaceutical interactions, and giving additional ethics lectures. She chairs the SLU Hospital ethics committee and has been an invited presenter for the SLU Center for Health Care Ethics and for Humanities in Medicine program at Washington University School of Medicine.

**Susan Chimonas, PhD**

[sc2254@columbia.edu](mailto:sc2254@columbia.edu)

Dr. Chimonas earned a PhD in sociology from the University of Michigan in 2000 and worked as a postdoctoral researcher at Rutgers University’s Institute for Health, Health Care Policy, and Aging Research. Se is a national expert in the field of physician-industry relationships and conflicts of interest in clinical care. She has written extensively about these topics in peer-reviewed journals and has played a critical role in the development of stronger conflict of interest policies at healthcare organizations around the country. She currently works at the Institute on Medicine as a Profession (IMAP) at Columbia University. While there, she has served on a task force which developed recommendations aimed at curbing conflicts of interest at academic medical centers, and another task force to develop equally rigorous standards for professional medical associations. She has developed tools and resources to promote ethical physician-industry relationships, including IMAP’s COI curriculum and best practices toolkits.

**Thomas L. Greaney, Esq**

[greanetl@slu.edu](mailto:greanetl@slu.edu)

Mr. Greaney received his JD from Harvard Law School in 1973, and joined Saint Louis University in 1987 after experience as a legislative assistant on Capitol Hill, a law clerk with the Federal Communications Commission, and a trial attorney in the Antitrust Division of the U.S. Department of Justice, where he became the assistant chief in charge of antitrust matters in healthcare. He currently serves as the Co-Director of the Center for Health Law Studies and Chester A. Myers Professor of Law at Saint Louis University School of Law. He is a nationally recognized expert on healthcare and antitrust law, and has produced an extensive body of scholarly writing on these topics in some of the country’s most prestigious legal and health policy journals.

**Himansh Khanna, MD**

[himansh.khanna@mssm.edu](mailto:himansh.khanna@mssm.edu)

Dr. Khanna earned his MD at the University of Cincinnati College of Medicine and completed training in urology at Maimonides Medical Center. He currently serves as a community urologist in New York. He practices medicine at Jersey Shore University Medical Center, Ocean Medical Center, and Southern Ocean Medical Center.

**Ron J. Levy, MHA**

rlevy3@slu.edu

Ron Levy has a long history of experience in healthcare systems delivery and financing. He served as president and CEO of SSM Health Care in Saint Louis, and then as director of Missouri’s Department of Social Services from January 2009 to August 2011. During that time, he served as a health policy advisor to Governor Jay Nixon and as the state’s health information technology coordinator. In 2011, the Missouri Hospital Association honored Levy with its Health Policy Leadership Award. He is currently the executive in residence at Saint Louis University School of Public Health. In this position, he continues to assist Governor Jay Nixon on special projects related to healthcare.

**Robert Lieberman, MD, MBA**

[roblieberman@gmail.com](mailto:roblieberman@gmail.com)

Dr. Lieberman completed an MD and MBA at Saint Louis University School of Medicine, and is currently a radiology resident at the University of Southern California. He completed an internship at Signature Medical Group in Saint Louis. His research interests include the intersection of business and health care.

**Tim McBride, PhD**

[tmcbride@wustl.edu](mailto:tmcbride@wustl.edu)

Dr. McBride received his PhD in economics from the University of Wisconsin and is Professor and Associate Dean for Public Health at the George Warren Brown School of Social Work at Washington University in Saint Louis. He has also served as a member of the nationally-recognized Rural Policy Research Institute (RUPRI) Rural Health Panel, which has provided frequent advise and briefings on rural health issues to the U.S. Congress and other policy makers for the last 15 years. Prior to joining Wash U, he was a Professor of Health Management and Policy at Saint Louis University, where he also served as Division Head of Health Policy, Co-Director of the Doctoral Program and Director of the Center for Health Policy Analysis. McBride’s research focus is on health economics, Medicare policy, the uninsured and insurance markets, rural health, state health policy, and long term care.

**Colin McCluney**

[eaf@amsa.org](mailto:eaf@amsa.org)

Mr. McCluney is currently in medical school the University of Washington School of Medicine. He has taken a year out of studying medicine to serve as the American Medical Student Association (AMSA’s) Education and Advocacy Fellow. There he has worked as a steering committee member and Co-Director of the Health Care for All Campaign.

**William Sage, MD, JD**

[wsage@law.utexas.edu](mailto:wsage@law.utexas.edu)

Dr. Sage received his JD and MD from Stanford University and currently serves as the Vice Provost for Health Affairs and the James R. Dougherty Chair for Faculty Excellence in Law at the University of Texas at Austin. He delivered

the third annual Bander Endowed Lecture at Saint Louis University and Washington University medical campuses in 2009 on conflicts of interest in medical research, education, and practice. He teaches health law, regulatory theory, antitrust and professional responsibility and has published in numerous areas, including medical malpractice, the economics of health care, disclosure laws, medical liability, health care reform, health policy, and professionalism.

**Julie Taitsman, MD, JD**

[paffairs@oig.hhs.gov](mailto:paffairs@oig.hhs.gov)

Dr. Taitsman received an MD from Brown University and a JD from Harvard Law School. Taitsman currently serves as the Chief Medical Officer for the Office of Inspector General for the U.S. Department of Health and Human Services. Previously at the OIG, Taitsman also served as Special Counsel for Health and Science to the Senate Finance Committee. Before joining the OIG, Taitsman practiced health care law in Washington, DC, focusing on Medicare payment and coverage and regulation of drugs, biologics, and medical devices.

**Jason S. Turner, PhD, MAE**

[jturne32@slu.edu](mailto:jturne32@slu.edu)

Turner received his PhD in Health Services Organization and Policy at the University of Michigan and is an Assistant Professor of Health Management and Policy at Saint Louis University School of Public Health. He also holds appointments at the Saint Louis University School of Law and the SLU Center for Outcomes Research, and is a fellow at the Academy of Healthcare Management. Dr. Turner has spent a number of years in the healthcare sector, first as a controller for a large, national healthcare insurer and administrator for a hospital chain. Now, he teaches corporate finance and accounting to graduate students in the MHA program with a focus on how finance and accounting relate to the overall strategy of healthcare institutions. He conducts research focused on capita structure and investment behavior, assessment of risk, the implementation of payment methodologies, and community benefit.

**Anji Wall, MD, PhD**

[anjiwall@gmail.com](mailto:anjiwall@gmail.com)

Dr. Wall is a recent graduate of Saint Louis University School of Medicine, where she achieved doctorates in both Medicine and Health Care Ethics. She is currently a general surgery resident at Vanderbilt University. She has authored a CME on free drug samples and several papers in bioethics on topics including research ethics, medical ethics in developing countries, organ donation, and ethics education. She is also the author of a forthcoming book entitled “Ethics for International Medicine: A Practical Guide for Medical Aid Workers in Developing Countries.” In addition, she is collaborating with the surgical ethics group at Washington University and the American College of Surgeons to create an online ethics curriculum for surgical residents.

**Dan E. Wiener, MD**

[dwiener@chpnet.org](mailto:dwiener@chpnet.org)

Dr. Wiener received his MD from Brown University School of Medicine and training in emergency medicine at Jacobi Hospital at the Albert Einstein College of Medicine. He currently is Chairman of Emergency Medicine at St. Luke’s-Roosevelt Hospital in New York. His research interests include emergency department overcrowding and patient flow, patient satisfaction, CQI and patient safety and disaster preparedness.

**Matthew Wynia, MD, MPH**

[matthew.wynia@ama-assn.org](mailto:matthew.wynia@ama-assn.org)

Dr. Wynia received his medical degree from Oregon Health and Science University school of Medicine and his MPH from Harvard School of Public Health. He is board certified in internal medicine, emergency medicine, and infectious diseases and practices medicine and is a Clinical Professor of Medicine at the University of Chicago. He is also the Director of the Institute of Ethics at the American Medical Association (AMA) and executive director of the Ethical Force Program. As director, he oversees a range of research projects on topics including physicians’ responses to utilization review and market pressures in medicine, physician professionalism and the role of professionals in society, ethics and access to care, codes of ethics, encouraging professional responsibility for trust in health care, and creating performance measures for health care ethics. He has over 125 published articles, book chapters, and reports.
