# Supplementary material for: Curricular priorities for business ethics in medical practice and research: recommendations from Delphi consensus panels
Source: BMC Med Educ. 2014 Nov 15;14:235. doi: 10.1186/1472-6920-14-235 (PMC4289297; doi:10.1186/1472-6920-14-235)
Supplement: Supplementary file 2 — Additional file 2: Business Ethics in Medical Research Panelists. (DOC 36 KB) [file 12909_2013_1100_MOESM2_ESM.doc]

**Appendix B: Business Ethics in Medical Research Panelists**

**Salvador Cruz-Flores, MD**

[**salvador.cruz-flores@ttuhsc.edu**](mailto:salvador.cruz-flores@ttuhsc.edu)

Dr. Cruz received his medical degree from the Universidad Autonoma de Nuevo Leon in Monterrey Mexico, where he also completed residencies in internal medicine and neurology. Dr. Cruz trained in neurology at Saint Louis University and completed a fellowship in cerebrovascular diseases and neurocritical care. He also completed an MPH at SLU in 2008. He also completed an intensive bioethics course at the Kennedy Institute of Ethics in 2009 and completed the Bander Center Fellowship at SLU in 2011. Dr. Cruz serve as Interim Chairman and Professor in the Department of Neurology at SLU, the Director of the Souers Stroke Institute, and Associate Director of the Neurology Residency Training Program. He is now Professor and Chairman of Neurology at Texas Tech University Health Sciences Center. Dr. Cruz has published numerous articles and presented abstracts on various topics related to cerebrovascular disease and neurocritical care.

**James DuBois, PhD, DSc**

[duboisjm@slu.edu](mailto:duboisjm@slu.edu)

Dr. DuBois received his PhD in philosophy at the International Academy of Philosophy in Liechtenstein and his DSc in psychology at the University of Vienna. From 2008-2013 he served as the inaugural Director of the Bander Center for Medical Business Ethics at Saint Louis University. He is currently Professor of Medicine at the Washington University School of Medicine, where he directs the Center for Clinical Research Ethics within their Institute for Clinical and Translational Science (ICTS). A journal he founded, *Narrative Inquiry in Bioethics: A Journal of Qualitative Research*, highlighted physician’s personal stories of living with conflicts of interest in one of its first issues. In addition to medical business ethics, his research interests include research ethics in mental health, preventing wrongdoing in healthcare, ethics of organ transplantation, moral development and education, and empirical research on ethical issues.

**Jesse Goldner, JD**

[goldneja@slu.edu](mailto:goldneja@slu.edu)

Mr. Goldner received his M.A. in psychology from Columbia University and his JD from Harvard Law School. He currently serves as the John D. Valentine Professor of Law at Saint Louis University School of Law. He has secondary appointments at Saint Louis University School of Medicine as Professor of Law in Psychiatry and Professor of Pediatrics, Departmental Adjunct Professor in the Department of Health Care Ethics, and Professor of Health Administration at the School of Public Health. His interests lie in law and psychiatry and law and medicine, focusing on the rights and welfare of research participants and research on human subjects. He was co-founder and director of the Center for Health Law Studies and co-edited the Journal of Health Law. He has chaired the Saint Louis University Institutional Review Board (IRB) and was initial chair of the Council on Accreditation of the Association for the Accreditation of Human Research.

**Cindy Kiel, JD**

[cmkiel@ucdavis.edu](mailto:cmkiel@ucdavis.edu)

Ms. Kiel received her JD from the University of Utah College of Law. She practiced law at Utah Legal Services and the law firm of McMurray, McMurray, Dale and Parkinson in the fields of consumer protection, government records access, education law, false claims act litigation, corporate transactional work, employment law, contract breach litigation, immigration, and intellectual property infringement. She later worked in the Sponsored Programs office at the University of Utah and obtained her Certification in Research Administration. In 2011, she became Executive Associate Vice Chancellor for Research Administration at UC Davis to provide leadership over research administration and compliance areas for the University. Previously, she served as Assistant Vice Chancellor for Research Services at Washington University in Saint Louis, overseeing grants and contracts, interdisciplinary research development and export controls. Prior to her work there, Kiel was Executive Director for Sponsored Projects at the University of Nevada, Reno with oversight over pre-award, post-award financial, research integrity, conflicts of interest and compliance. She is an active member in the Council on Government Relations and speaks nationally on global research, research compliance, proposal development, Federal contracting, subcontracts, budgeting and cost-share, negotiation tactics, export trade controls technology transfer, and research infrastructure. She reviews proposals for the NIH and the DOD.

**Terry May, PhD**

[mayte@egr.msu.edu](mailto:mayte@egr.msu.edu)

Dr. May received his PhD in Environmental Biology at the University of Colorado. His general interest in the process of research led him to undertake a career in research administration spanning nearly 26 years at the University of Maine, the University of Delaware, Thomas Jefferson University, and Northern Arizona University. May moved to Michigan State University in 1998 and is currently the Faculty Conflict of Interest Information Officer. His efforts there have focused on implementing the university’s faculty conflict of interest policy, supporting a task force of senior faculty considering research mentoring of graduate students, and being the Series Coordinator for Graduate Education and Postdoctoral Training workshops in the responsible conduct of research.

**Genevieve Pham-Kanter, PhD**

[genevieve.pham-kanter@ucdenver.edu](mailto:genevieve.pham-kanter@ucdenver.edu)

Dr. Pham-Kanter received her PhD in economics from the University of Chicago, an MPhil from Cambridge University, and an MS in Tropical Public Health from Harvard University. She also completed a postdoctoral research fellowship at Princeton University at the Woodrow Wilson School of Public and International Affairs, office of Populations Research and Center for Health and Wellbeing. She is currently an Affiliated Assistant Professor in the Department of Economics at Colorado University. Her areas of expertise include health economics, labor economics, social economics and applied microeconomics.

**Heather Pierce, JD, MPH**

[hpierce@aamc.org](mailto:aamcpresident@aamc.org)

Ms. Pierce received her JD at New York University and her MPH at Boston University. She currently serves as the Association of American Medical Colleges (AAMC)’s Senior Director for Science Policy and Regulatory Council in Scientific Affairs. She is a leading scholar on many issues, including conflicts of interest, human subjects protection, compliance and other regulatory issues related broadly to biomedical research. She is part of the Scientific Affairs Senior Leadership team. Ms. Pierce’s accomplishments include working as an Associate in the Health Care Group at Ropes & Gray in New York. Her practice focused on regulatory compliance issues including research with human subjects, medical information privacy and security, and fraud and abuse counseling. She worked with a variety of clients, including hospitals, universities, academic medical centers, and pharmaceutical companies. She has assisted with state and federal government investigations and the development of compliance and training programs.

**Victoria K. Shanmugam, MD**

[VKS4@gunet.georgetown.edu](mailto:VKS4@gunet.georgetown.edu)

Dr. Shanmugam received her medical doctorate at the Imperial College School of Medicine in England. She was a member of the Royal College of Physicians before moving to Washington D.C. and completing residency and fellowship training at Georgetown University Hospital, where she later joined the Division of Rheumatology. She now serves as an Assistant Professor in the Division of Rheumatology, Immunology & Allergy in the Department of Medicine at Georgetown University, and as a KL2 scholar at the Georgetown-Howard Universities Center for Clinical and Translational Science. She specializes in a variety of rheumatological and immunological disorders, including rhematorid arthritis, schleroderma, inflammatory arthritis, and vasculitis. Her research has prompted many awards, including an American College of Rheumatology Physician Scientist Development Award, the John Eisenbery Memorial Career Development Award, and the C. Bertram Hoffberger Research Achievement Award.

**Raymond Tait, PhD**

[taitrc@slu.edu](mailto:taitrc@slu.edu)

Dr. Tait received a doctorate in clinical psychology from the University of Illinois at Chicago Circle. He completed post-doctoral training in pain and behavioral medicine at the University of Virginia, and then joined the faculty of Saint Louis University. He is a Professor in the Department of Neurology & Psychiatry and holds an adjunct appointment in the Center for Health Care Ethics. He has served as director of research in the Department of Psychiatry, chair of the biomedical IRB, and the Research Planning Committee for the School of Medicine. He is currently administrative chair for both the biomedical and behavioral and social science IRBs. He is a long-time member of the International Association for the Study of Pain and of the American Pain Society (APS), and has been a governor’s appointment to the Missouri Advisory Council on Pain and Symptom Management since 2003 and served on the board of directors of the Missouri Pain Initiative during the same period. He specializes in pain and healthcare disparities research, and has developed an interest in research ethics related to vulnerable populations.

**Julie Taitsman, MD, JD**

[paffairs@oig.hhs.gov](mailto:paffairs@oig.hhs.gov)

Dr. Taitsman received an MD from Brown University and a JD from Harvard Law School. Taitsman currently serves as the Chief Medical Officer for the Office of Inspector General for the U.S. Department of Health and Human Services. Previously at the OIG, Taitsman also served as Special Counsel for Health and Science to the Senate Finance Committee. Before joining the OIG, Taitsman practiced health care law in Washington, DC, focusing on Medicare payment and coverage and regulation of drugs, biologics, and medical devices.

**Patricia M. Tereskerz, JD, PhD**

[pjm7s@virginia.edu](mailto:pjm7s@virginia.edu)

Dr. Tereskerz received her JD and PhD from the University of Virginia and serves as an Associate Professor of Medical Education at the University of Virginia School of Medicine. There, she also Directs the Program in Ethics and Policy in Healthcare. She is an attorney and epidemiologist specializing in ethics and healthcare policy, and her research focuses primarily on financial conflicts of interest in research, clinical research law and ethics, and patent law as it relates to public policy and ethics. She is the founding chair of the University of Virginia’s Conflicts of Interest Committee.

**David K. Warren, MD, MPH**

[dwarren@dom.wustl.edu](mailto:dwarren@dom.wustl.edu)

Dr. Warren received his MD from the University of Pittsburgh School of Medicine and training in internal medicine and infectious diseases at Washington University. He received his MPH at Saint Louis University in 2005. He is currently is an Associate Professor of Internal Medicine in the Division of Infectious Diseases at Washington University School of Medicine and a infectious disease specialist and epidemiologist at Barnes-Jewish Hospital in Saint Louis. He serves as medical director for infection prevention at Barnes Jewish Hospital. His research interests include epidemiology and prevention of nosocomial infections. He directs the Masters of Science in Clinical Investigation program within the Clinical Research Training Center at Washington University School of Medicine.
